# A Novel Cooperative AI-Based Fall Risk Prediction Model for Older Adults

**DOI:** 10.3390/s25133991

**Published:** 2025-06-26

**Authors:** Deepika Mohan, Peter Han Joo Chong, Jairo Gutierrez

**Affiliations:** 1 Department of Electrical and Electronic Engineering, Auckland University of Technology, Auckland 1010, New Zealand; deepika.mohan@aut.ac.nz; 2 Department of Computer and Information Sciences, Auckland University of Technology, Auckland 1010, New Zealand; jairo.gutierrez@aut.ac.nz

**Keywords:** fall risk prediction, fuzzy logic, deep belief networks, meta-model, random forest, vital signs, ADLs

## Abstract

**Highlights:**

**What are the main findings?**

- A cooperative AI-based meta-model combining Fuzzy Logic and Deep Belief Networks achieved 90% accuracy, 100% specificity, and 85.71% sensitivity in predicting future fall risk in older adults.
- The model outperformed traditional fall risk assessment tools by utilizing daily activity patterns and vital sign data for personalized prediction.

**What is the implication of the main finding?**

- Enables early prediction of fall risk to support timely interventions and improve safety among the elderly population.
- Offers a smart, scalable, and non-intrusive solution for continuous health monitoring and fall risk prediction in real-world settings.

**Abstract:**

Older adults make up about 12% of the public sector, primary care, and hospital use and represent a large proportion of the users of healthcare services. Older people are also more vulnerable to serious injury from unexpected falls due to tripping, slipping, or illness. This underscores the immediate necessity of stable and cost-effective e-health technologies in maintaining independent living. Artificial intelligence (AI) and machine learning (ML) offer promising solutions for early fall prediction and continuous health monitoring. This paper introduces a novel cooperative AI model that forecasts the risk of future falls in the elderly based on behavioral and health abnormalities. Two AI models’ predictions are combined to produce accurate predictions: The ${AI}_{1}$ model is based on vital signs using Fuzzy Logic, and the ${AI}_{2}$ model is based on Activities of Daily Living (ADLs) using a Deep Belief Network (DBN). A meta-model then combines the outputs to generate a total fall risk prediction. The results show 85.71% sensitivity, 100% specificity, and 90.00% prediction accuracy when compared to the Morse Falls Scale (MFS). This emphasizes how deep learning-based cooperative systems can improve well-being for older adults living alone, facilitate more precise fall risk assessment, and improve preventive care.

## 1. Introduction

Falling is a predominant cause of pain, disability, loss of functioning, and untimely mortality in the older population. As reported by the World Health Organization (WHO) [1], approximately 28–35% of individuals aged 65 years and above have at least one fall each year, increasing to 32–42% in individuals aged over 70. The economic burden of injury resulting from falls is significant and increasing globally, not just for older adults but also for their families and the wider community. In New Zealand, nearly one in three people aged over 65 experience a fall every year [2]. In recent Waipapa Taumata Rau [3], University of Auckland research, Accident Compensation Corporation (ACC) data from the Bay of Plenty and Lakes Taupō and Rotorua districts were compared over five years (2014–2018). Falls were the most common cause of injury for older Māori, and most of these occurred in the home. Out of almost 150,000 total claims, around 9000 were from Māori over 50 years of age, and 55,000 were from older non-Māori. The rate of claims for non-Māori was 46% higher than for Māori. In 2024, ACC NZ had approximately 189,407 current claims for falls injury for individuals aged 60 and above [4].

Falls in older adults tend to result in serious injuries, and in some cases, death [5]. Having fallen can also lead to loss of confidence, which leads people to avoid doing things for fear of falling again. This loss of confidence can lead to a worsening of physical function, creating a cycle that contributes further to fall risk and decreased quality of life [6]. Preventing activities weakens muscle strength and balance and predisposes one to subsequent falls. However, ongoing physical exercise after a fall is recommended since it produces strength, improves balance and coordination, and adapts essential health measures such as blood pressure, blood glucose, and body weight. Preventions are therefore important in reducing the prevalence and severity of falls, especially those employing interventions that combine health-oriented solutions with environmental preventive interventions.

Fall prediction [7] is also vital in that it assists in contributing to safety by assessing the risk of falling for a person based on physical activity levels, medical conditions, balance, and environmental hazards. Statistical computation and machine learning algorithms are used to detect patterns and predictors of falls by predictive models. According to [8], the conventional monitoring systems are generally too slow or inaccurate for timely intervention. Instead, artificial intelligence (AI) and machine learning (ML) offer good alternatives through significant enhancements in fall prediction systems. AI and ML improve data processing capabilities, enable real-time monitoring, aid adaptive learning, and enable integration with smart systems. The integration of AI and ML in fall prediction brings accuracy and adaptability in the process, which are not achievable through traditional methods. By facilitating ongoing learning and fine-tuning, such technologies enable more precise fall risk assessment and interventions earlier on, which, in the long run, results in enhanced safety and quality of life of the elderly [9]. With the ongoing development of technologies, the promise of such technologies to construct safer and more autonomous living for the elderly will expand.

The conventional assessment for fall risk has traditionally hinged on functional testing and clinical opinion that, while helpful, lack predictive capacity and necessitate subsequent testing. For example, the research study presented in [10], with the Irish Longitudinal Study on Ageing (TILDA) dataset has demonstrated that AI models can predict simple and complex falls with good accuracy. However, there are issues in making the results more generalizable, especially for complex fall events that are tied to severe health conditions. Further, Parkison’s disease research [11] has ascertained that disease-specific and functional measurements are the key risk predictors for falls, highlighting general evaluation strategies involving clinical assessments along with artificial intelligence-based methodologies. Even though all these strides have been taken, current models usually lack the applicability required for real-world problems, so there is a need to create stronger predictive models.

Our developed research model introduces a novel cooperative AI model that can predict future falls in the elderly with higher accuracy and validity. To our knowledge, the present study in this paper is the first scientific investigation to specifically use two different AI models of different dimensions embedded with the meta-model to predict the probability of future falls among older adults. We first calibrate this model with data downloaded from public repositories. In the following phase (future work), after validation, we plan to incorporate real-time information from older adults. Utilizing multi-modal datasets like physiological, behavioral, and functional assessments, the model aims to bypass the limitations of traditional classifiers and improve fall risk stratification. The strategy built has initially been validated through public datasets to ensure stability before incorporating real-time information in future stages. The proposed AI-driven solution can potentially enable early interventions, decrease the likelihood of fall injuries, and enable independent living for the elderly. As newer AI and ML technologies advance, they offer a robust platform to enhance fall forecasting to achieve enhanced healthcare outcomes. The remainder of the paper is structured as follows: Section 2 discusses a review of related work, Section 3 provides the proposed methodology, Section 4 describes the experimental results, Section 5 includes the discussion, and Section 6 summarizes the conclusions and indicates future research directions.

## 2. Literature Review

The absence of real-time monitoring, tracking, and data transmission aggravates the issues of fall risk management in elderly individuals in healthcare facilities [12]. In the vast majority of fall detection and prediction studies, the following criteria were lacking from either the developed model or the study background:

- The models that were developed are not all able to handle multiple profiles.
- At times, the pre-trained model is restricted to understanding only a particular dataset. When there is a change, it fails to learn or provide an output based on the changes.
- Most of the deep learning techniques used in the system model for fall prediction are not transparent, are uncontrollable, and tend to be opaque.

These problems call for innovative approaches to the design of healthcare monitoring systems, which must be able to function under restricted resources, e.g., system performance, and respond dynamically to the fall incident. Hence, this section will strive to conduct an extensive literature review to shed light on the key aspects of developing a flexible and uncontrollable monitoring system for fall prediction and prevention. This section tries to give a comprehensive overview of the upcoming technologies, like AI- and motion-based predictive models, IoT devices, non-wearable and wearable technology, data mining, and real-time analytics procedures for the dynamic world. Most of this literature review discusses the methodologies used in the prediction of falls and the assumptions obtained through motion detection, machine learning, deep learning, and other rule systems. Multidimensional models for the prediction of fall and identification of risk factors that are challenging when used by elderly people are also identified. Also, the performance of AI-IoT-based models is compared with that of non-AI, traditional models to show that the AI-inclusive systems outperform the non-AI systems in terms of performance, accuracy, and efficacy.

The IoT and AI represent novel solutions towards fall prediction and prevention in elderly individuals, enhancing assisted living (AL) and healthcare monitoring (HM) services through the computation of their routines. A study [13] focused on predicting fall danger in long-term nursing home patients using advanced RNN models like Long Short-Term Memory (LSTM) and Gated Recurrent Units (GRUs). The models were calibrated using the Long-Term Care Minimum Dataset (MDS) 3.0 and prescription drug claims from five facilities in Western Pennsylvania. The models forecasted falls within 90 days of the completion of an MDS assessment and were compared with a standard classification and regression tree–logistic regression (CART-logit) model. The RNN, LSTM, and GRU models performed similarly, with an area under the receiver operating characteristic curve (AUROC) of approximately 0.74, better than that of the CART-logit model (AUROC = 0.67). This study points out the strength of RNN models in leveraging sequential data to improve fall risk prediction. It also refers to limitations, including the nature of neural networks, which complicate clinical uptake due to low transparency in decision-making. Despite such constraints, this study reveals the potential of RNN-based models to enhance fall prediction and optimize healthcare resource utilization in nursing homes.

Another study [14] investigated how to use artificial intelligence (AI) to foresee the risk of falling among elders based on examining patterns of walking with computer vision and machine learning techniques. Stride time, step time, cadence, and stance time were the extracted gait features based on information from two different sources, which were the MMU-FRiP dataset and the Mendeley-provided public dataset. The MMU-FRiP dataset included 21 young adults, and the Mendeley dataset included 44 older adults with increased fall risk according to the Performance-Oriented Mobility Assessment (POMA). Two experimental settings were explored: analyzing gait parameters separately per foot and averaging across both feet. Twelve classification algorithms were employed, including State Vector Machine (SVM), Decision Tree (DT), Random Forest (RF), Light Gradient-Boosting Machine (LightGBM), XGBoost, CatBoost, AdaBoost, K-Nearest Neighbors (KNN), Voting, Naïve Bayes (NB), Multilayer Perceptron (MLP), and Bagging. Light Gradient-Boosting Machine (LightGBM) was the best-performing model, with high accuracy at 96% and improved computational performance and predictive ability over other machine learning models. While this study had promising results, it had limitations, including a small, non-homogeneous dataset and the utilization of synthetic data, which impaired the generalizability of the model and increased the risk of overfitting. This study emphasizes the potential of AI to improve fall risk predictions and population health interventions but mentions challenges in applying these models to clinical real-world scenarios.

A detailed and extensive review [10] examined the use of machine learning in the guise of Random Forests and Explainable Artificial Intelligence (XAI), for the prediction of different types of falls in older persons. This study was grounded on data collected from the Irish Longitudinal Study on Ageing (TILDA) with a focus on 46 variables, including a frailty index (Syncope–Falls Index, SYFI) for assessing health deficits in falls and syncope. The data was analyzed with four Random Forest models for predicting simple, complex falls, and syncope based on outcomes measured as falls reported within 6 years. This study found that simple falls were more a function of accident and negative contributing factors for frailty, and complex falls in relation to more serious health states. Although the models were very accurate, particularly for uncomplicated falls and syncope (up to 83%), they were not perfect, e.g., moderate performance on complicated falls. This research demonstrates the potential of AI to predict fall risk but indicates that further work is necessary to enhance model generalizability and further understand the relationship between the features and types of falls.

A study [15] aimed to establish a legitimate set of functional and disease-specific tests that are predictive of fall risk in patients with early-stage Parkinson’s disease (PD). This addresses the ongoing issue of how to target those patients at most risk despite optimal medication. The investigation was conducted on 101 participants who undertook a set of tests that comprised the Tinetti, Berg, Timed Up and Go, Functional Reach, and the Physiological Profile Assessment. Within 6 months, 48% of the participants had suffered at least one fall, while 24% had suffered recurrent falls. The study found that the composite of several tests, such as the Unified Parkinson’s Disease Rating Scale (UPDRS), Tinetti total score, and postural sway, yielded the best predictive value (78% sensitivity and 84% specificity). The findings show that a battery of disease-specific tests and balance measures can effectively predict the risk of falling in early-stage PD patients. In essence, this study highlights the necessity of using both generic disease measures and specific functional tests in fall prediction, which can eventually lead to more suitably targeted interventions for PD patients at risk of falling.

A similar study [11] tried to find predictors of falls in individuals with Parkinson’s disease (PD). The investigation involved 58 participants with first-stage PD and followed them up at baseline and 3.5 years. Relevant predictors were a history of falls, gait impairment, cognition status (Mini-Mental State Exam), and other predictors, including freezing of gait and pain. The authors found that the history of falls was a strong short-term predictor of subsequent 6-month falls. Long-term predictors were tandem gait abnormality and lower baseline cognitive test scores. The study was also plagued by problems of participant dropout and measurement instrument coarseness, which were not sensitive to mild impairment in some cases. Despite these limitations, the findings have high potential for fall prediction in individuals with PD and show that both the measurement of mobility and cognition are important in the prediction of falls. Based on background studies performed, it can be realized that the falls can be predicted among older people with appropriate approaches like monitoring of vital signs, Activities of Daily Living (ADLs) evaluation, observing behavior, emotional analysis, and posture monitoring.

The challenges, issues, and limitations of fall prevention are targeted towards the deployment, flexibility, and profitability functions. Older adults must be educated to embrace the models developed, and adequate flexibility must be imposed. Moreover, it is unrealistic to anticipate that older adults to always use smart devices to move around in the smart environment. It has been a critical issue within the dataset to analyze the issues before the construction of the model. Deep learning has progressed dramatically, as much as accuracy is involved in domains such as behavior, health, image recognition, object detection, and other emerging technologies. Human-less AI is penetrating numerous businesses, although it still exists in the development phase for security purposes. As AI is being trained from the environment and its actions through experience with the environment, there are several factors to consider. While applying the concept of aided robots for surveillance of patients and preventing falls, judgment and identification through these parameters, with the use of AI and robots at times, may forecast erroneous outcomes.

In [16], an autonomous mobile assistant model was suggested that uses Deep Reinforcement Learning to interlink the nursing environment and patients, providing allocation, discharge summary, and fall monitoring; however, the model interrelated the details of inpatients and outpatients. Even though the model employs deep learning to identify its environment, it never achieves a 100% identification rate. However, after applying risk extraction and reduction techniques, AI can be applied in any relevant subject, e.g., safety. Early fall prediction using AI technology combined with IoT can initiate an on-time alarm and alert for emergency assistance, particularly for the elderly who live alone. Elderly and their families can feel more at ease by monitoring older adults’ external and cognitive well-being. Through the examination of reports collected from distant health centers with the use of AI, older persons can develop confidence in difficult situations. With the implementation of temporal convolutional network models and deep learning models, more accurate and dependable predictions can be attained. Hence, AI-IoT-based monitoring may be defined as a new generation in the medical field for disease prediction, detection, prevention, and emergency alarm triggering, particularly for fall prediction among elderly people. Nevertheless, to create a comprehensible and credible AI system, greater effort and research must be devoted to real-time monitoring. These approaches, when integrated with AI and ML technologies, represent a powerful set of tools for predicting falls among older adults. AI and ML algorithms can screen large amounts of data from such monitoring systems to provide more precise and reliable estimates of fall risk. Studies on predictive modeling for falls [17] and AI-based healthcare applications [18] have indicated the enhanced ability of such technology to enhance care and foster safety among older adults.

Our research is evidently aimed at the prediction of future falls among older adults based on vital signs and ADL monitoring. Our meta-model is easily integrated with the suggested cooperative AI model based on vital sign and ADL data, with very precise outcomes and proximity to the ground truth obtained by the application of the MFS. This proposed work is an extension of our previous works, where our first model used only vital signs to predict the risk of future falls in the elderly [19] and our second model used only ADLs to predict the risk of future falls. Now, in this proposed work, we are going to combine our two collaborative AI models to mix with the meta-model and the ground truth, which is the MFS, to produce the final fall risk prediction output. This new approach equips our model with the ability to create timely alerts for fall prediction in older adults using real-time data, which is discussed in the future work section of our research.

## 3. Proposed Model

This model is utilized to predict impending falls and classify fall risk levels in older adults aged 60 years and older. Based on the assessment by the AI-driven Cooperative Meta-Model, risks of falls were categorized into Low, Moderate, and High. The prediction output is derived through the cooperation between two AI models. Initially, the data of older adults is sourced from a public database for model training and testing. Section 3.1 gives an overview of the process of fall prediction through vital signs and Fuzzy Logic, Section 3.2 describes the process through Activities of Daily Living (ADLs) and a Deep Belief Network (DBN), and Section 3.3 provides the overall final model prediction for the cooperative AI model.

### 3.1. Fuzzy-Based Fall Risk Prediction System

**Figure 1:**
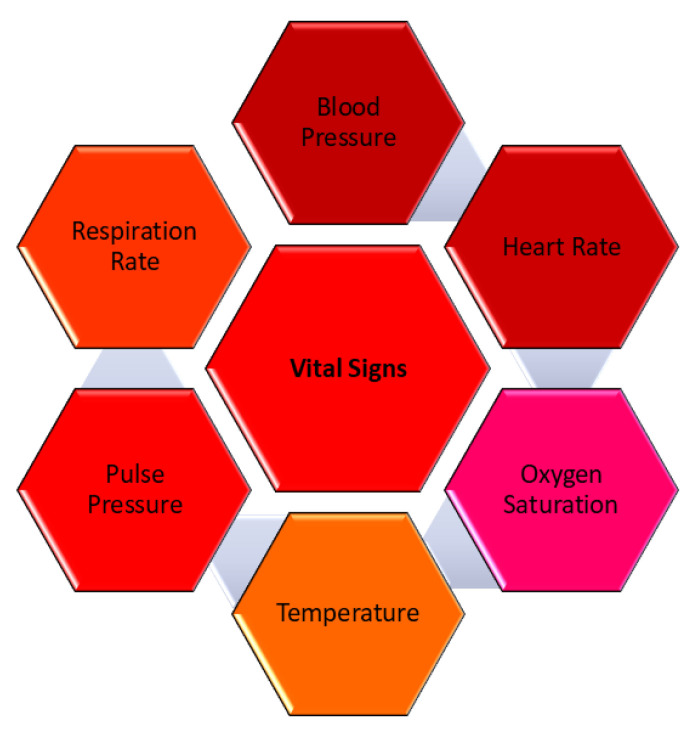
Vital sign parameters.

Our first AI model, described in [20], introduces an innovative approach to predicting fall risks in elderly individuals by analyzing vital signs such as the heart rate, blood pressure, and blood oxygen levels using Fuzzy Logic. The combination of fall risk factors and vital sign monitoring is critical for fall prediction. Vital signs are important indications of a person’s overall health and current medical status. It is one of the most significant and sensitive parameters for healthy living that moves with an individual’s lifestyle; therefore, it is vital to monitor them regularly, especially in older adults, as any imbalance in them may lead to a fall. It is logical that the higher the frequency of vital sign measurements, the faster clinical deterioration is noticed [21]. According to [22], the earliest evidence of potentially dangerous physiological changes or disruptions in the body can frequently be discovered in vital signs, which can also serve as the first indication that the disease has stabilized. The four most significant and conventional vital indicators are blood pressure (BP), temperature, pulse, and respiration rate. Recent additions include pain, threshold, and oxygen saturation measures [23]. Figure 1 describes the vital sign parameters. It is possible to anticipate future falls and reduce the risk of falls if all these vital signs are regularly checked and compared to the individual’s medical history. As a result, the first model developed in [20] focuses on monitoring blood pressure, heart rate, and blood oxygen levels to anticipate and forecast a potential fall in older adults.

Initially, data for this study were sourced from eICU Collaborative Research Database Demo 2.0.1-PhysioNet, a public repository, to verify the model’s accuracy [24]. The data obtained from the public repository is separated into fallers and non-fallers, and each type is sent independently to the fuzzy model. The fuzzy prediction model gathers vital sign data such as blood pressure, pulse pressure, heart rate, and blood oxygen saturation. Using the membership function and fuzzy rules, the model classifies the risk of falling as Normal, Low, Moderate, High, or Emergency. This method focuses on identifying fall risks in real-time, aiming to mitigate injuries or fatalities associated with falls in older adults. The system collects data from three distinct sources, which are processed and evaluated against the MFS for validation. The aim of the proposed Fuzzy-Based Fall Prediction Model (${AI}_{1}$ model) is to identify elderly individuals at risk of falls so that the economic and personal burden relating to injuries caused by falls can be minimized. This developed model would be a very good companion for older people, possibly those who live alone, as it educates them about falling risks and the prevention of any further falls. The model focuses on continuous monitoring of vital signs (blood pressure/heart rate/blood oxygenation) for early detection of fall-related abnormalities. Integrating this model into smartwatches for real-time monitoring can alert the elderly and their caregiver in most cases, enhancing proactive fall prevention. One of the advantages of the proposed model is the accuracy level of 95.24% with 100% specificity and 93.75% sensitivity with respect to the MFS using data from three sources. A key advantage of this approach is its exclusive use of vital signs for prediction, coupled with its high accuracy compared to the MFS, and it classifies fall risk into five levels: Normal, Low, Moderate, High, and Emergency. These findings underscore the effectiveness of the ${AI}_{1}$ model in enhancing fall prediction strategies for the elderly. The in-depth detail of the fuzzy model and the fall prediction algorithms, along with the fuzzy rule table, are elaborated in the Appendix A, Appendix A.1.

### 3.2. DBN-Based Fall Risk Prediction System

The development of advanced monitoring techniques, as well as the prediction and prevention of falls in the elderly population, has been the primary focus of recent research. High-performance fall prediction requires a comprehensive understanding of key features such as gait measurements, balance, muscle strength, and environmental factors. According to a study [25], 50–80% of patients admitted to emergency departments for falls with injuries identify environmental home risks as the cause of their falls. Determining these indicators, along with the integration of wearable sensor data, medical history, and demographic data, can significantly improve predictive precision. This section of the paper elaborates on the development of an intelligent fall prediction model that forecasts future falls among the elderly by continuously monitoring their Activities of Daily Living (ADLs) and detecting abnormalities [26]. To demonstrate the capabilities of deep learning to anticipate early fall risk, the model is built on a Deep Belief Network (DBN) and uses advanced AI techniques such as contrastive divergence for pre-training, backpropagation for fine-tuning, and the Adam Optimizer for minimizing loss. This could lead to more prompt treatment, lowering the frequency and severity of falls among the elderly.

The ${AI}_{2\;}$ model monitors ADLs, including actions like sitting, standing, walking, running, and jumping, to detect abnormalities through continuous observation. Evaluation of the proposed model is achieved by comparing prediction outcomes with traditional fall prediction techniques and ground truth (GT). It leverages the Long-term Movement Monitoring Database Version: 1.0.0 from PhysioNet [27], which comprises data from 71 elderly community residents continuously monitored over three days (75 h) using a 3D accelerometer (Analog Devices ADXL330; range ±3 g; 300 mV/g at the output). This dataset analyzed participants’ gait, stability, and fall risk. These findings demonstrate that contemporary deep learning technology can be effectively used to improve earlier fall risk prediction, hence reducing the likelihood and severity of falls among older adults. The DBN’s success exemplifies AI’s transformative potential in elder care, enabling autonomy and optimizing quality of life. The extraordinarily high sensitivity and specificity rates represent significant advancements over prior fall prediction methodologies, with this technology providing a useful aid to fall prediction. Fall risks are classified as Low, Moderate, and High.

To our knowledge, this is the first model that employs a Deep Belief Network (DBN) to predict falls in older adults using only ADLs and medical history. Each ADL is weighted according to its significance in fall prediction: sitting (0.2), standing (0.4), walking (0.6), running (0.8), and jumping (1.0). These weighted inputs are evaluated using the developed DBN-based Fall Risk Prediction Algorithm (DBN-FRPA). Pre-training and fine-tuning of the DBN model are performed using several Restricted Boltzmann Machine (RBM) architectures. The model’s evaluation against the Morse Fall Scale (MFS) demonstrates strong predictive capabilities, achieving an accuracy of 93.33%, a specificity of 100%, and a sensitivity of 92.86%. These findings underscore the effectiveness of advanced deep learning methodologies in predicting fall risks and assessing severity, thereby promoting fall prediction and enhancing safety for older adults. The detail of the DBN-based fall risk prediction model and the DBN algorithm pseudocode, along with the workflow flow, is elaborated in Appendix A, Appendix A.2.

### 3.3. AI-Based Cooperative Fall Risk Prediction System

Initially, in building our third model, we planned to use different types of comparators to combine the outcome of ${AI}_{1}$ and ${AI}_{2}$ to produce the future fall risk prediction output in the elderly. Certain types of systematic comparison were given thought, such as Weighted Average or Score Fusion, the Rule-Based Fusion Method, and the decision tree-based approach. Since there are major defects in all discussed fusion models, e.g., they have little ability to forecast outputs from training data, we explored other models utilizing both the training and the testing datasets. In our fall risk prediction meta-model-based system that we put forward, both the ${AI}_{1}\;\;$ and the ${AI}_{2\;}$ output values act as input features to train a combined model in our meta-model-based fall risk prediction system for generating a prediction. Here, we use a Random Forest algorithm for training our meta-model in this initial step. The Cooperative Meta-Model fall risk system algorithm is designed through the combination of outputs for ${AI}_{1}\;\;$ and ${AI}_{2}$ models.

#### Comparator Analysis:

**Figure 2:**
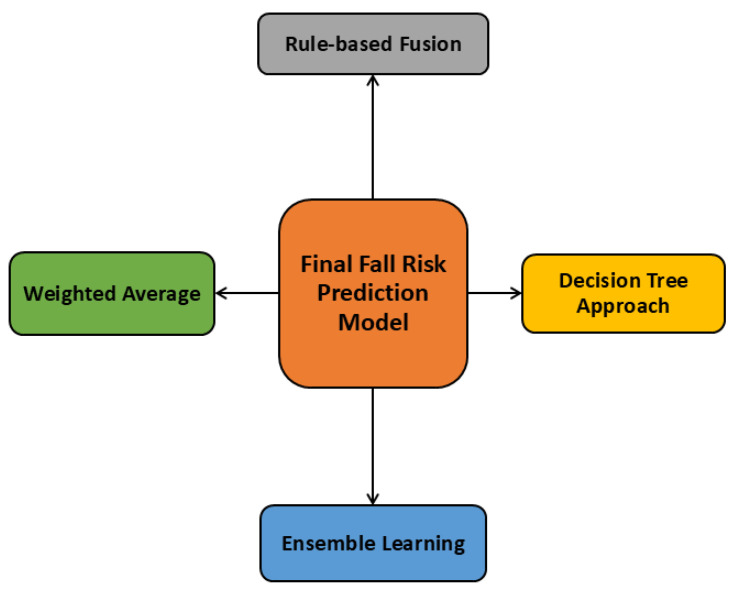
Comparator analysis.

To integrate two AI models, our initial intuition was to use comparators. In developing the third model, we aimed to utilize a series of comparators to effectively combine ${AI}_{1}$ (fuzzy-based fall risk prediction model), and ${AI}_{2}$ (DBN-based fall risk prediction model) results, ultimately resulting in an improved prediction of future fall risk among older adults. Several systematic comparison methods were investigated for this goal, such as the Weighted Average method [28], Rule-based Fusion Method [29], the decision tree approach [30], and ensemble learning [31]. Figure 2 depicts the comparator analysis.

Weighted Average or Score Fusion: In this comparison approach, the outputs of both models are normalized to a shared numerical range, and weighted averaging is employed to aggregate the results, as presented in (1).

(1) $$\mathrm{Final}\;\mathrm{prediction}=\frac{{AI}_{1}+{AI}_{2}}{2}$$

${AI}_{1}$ weight allocations are as follows: Normal → 0.2, Low → 0.4, Moderate → 0.6, High → 0.8, and Emergency → 1.0. ${AI}_{2}$ corresponding weights are as follows: Low → 0.33, Moderate → 0.66, and High → 0.99. Table 1 shows the weight assignment for the ${AI}_{1}$ and ${AI}_{2}$ models. The overall fall risk score is calculated based on the mean of ${AI}_{1}$ and ${AI}_{2}$ scores. The global weight distribution utilized to compute the final fall risk prediction result is illustrated in Table 2, derived from the score fusion measures. For example, if ${AI}_{1}$ predicts a result of 0.4 (*Low risk of fall*) and ${AI}_{2}$ predicts a result of 0.66 (*Moderate risk of fall*), then the ultimate comparator result would be (0.4 + 0.66)/2 = 0.53, “*Moderate fall risk*”. Although such a method is straightforward, it does not depict the complexity of fall risk estimation. Weighing and averaging scores in such a manner may be too simplistic and fail to provide a real representation of actual risk. Since this method is not good enough to capture the reasonableness of the resulting prediction based on two well-established models, we investigated the next comparator (Rule-based Fusion Method).

**Figure 3:**
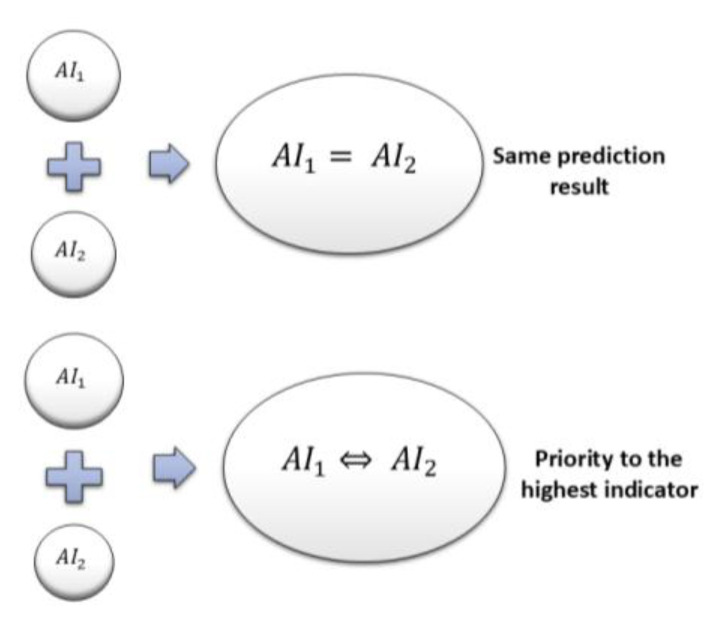
Final predictions derived from the Rule-Based Fusion Method.

Rule-based Fusion Method: In this approach, individual or threshold rules are applied to combine ${AI}_{1}\;$ and ${AI}_{2}\;$ prediction indicators. A majority voting approach is employed when both ${AI}_{1}\;$ and ${AI}_{2}$ predict the same category, and it is chosen as the final output. Priority-based rules are also stored to give preference to higher-level predictions over others. For example, if ${AI}_{1}\;$ shows “*Emergency*”, and ${AI}_{2}\;$ refers to a “*High risk of fall*”, the final inference will always be “*Emergency*”, regardless of what the second model reports. Likewise, if ${AI}_{1}\;$ shows “*Moderate risk of fall*”, and ${AI}_{2\;}$ suggests “*High risk of fall*”, the final inference will accordingly be “*High risk of fall*”, as this constitutes the greater risk factor. Figure 3 presents the final predictions derived from this rule-based method. While this method successfully orders instances by severity, it is possible that it fails to represent all situations correctly and might result in false alarms. Acknowledging these shortcomings, we further investigated another approach based on a decision tree comparator.

Decision-tree Approach: In this approach, the final fall risk estimation is performed based on a structured decision tree using the outputs of ${AI}_{1}\;$ and ${AI}_{2}\;$ logically to reach an informed decision. The biggest difference between this approach and the rule-based approach is that the decision tree is hierarchical rather than employing static priority-based rules. Table 3 provides the prediction of the decision tree approach. For both the rule-based and decision tree approaches, the analysis is purely performed using the prediction indicators (risk levels), and no weights are assigned to the fall risk levels.

“*Moderate-High*” or “*Low-Moderate*” may be an adaptive choice here instead of a rigid hierarchy. The tree can also incorporate weight updates with real-world information. Both methods integrate ${AI}_{1}$ and ${AI}_{2}$ predictions, but the decision tree method gives a more formalized and adaptable classification instead of strict rule-based priority choices. It is more adaptable and reduces misclassification from predetermined rules. Even though the model produces good predictions compared to the above two methods, it is still not convincing to use this analysis as the comparator cannot learn from the past and present predictions. Hence, we investigated the next learning model, which is ensemble learning.

Ensemble Learning: Ensemble learning is a powerful technique that integrates multiple models to improve prediction accuracy. Based on the background study conducted in [32,33,34] using ensemble learning, we intend to utilize this method for the final fall risk prediction analysis. In this paper, we implement a meta-classifier that takes ${AI}_{1}\;$ and ${AI}_{2}\;$ predictions as input features and produces one prediction. In contrast to conventional fusion techniques based on rule-based or weighted averaging techniques, ensemble learning adapts to the final prediction using training data dynamically. The techniques, such as weighted averaging, rule-based systems, and decision-tree techniques, incorporated inherent inflexibility and assumptions that restricted their flexibility. Those methods did not assist in improving their forecasting ability during the specified timeframe, mainly because they had no training program.

The meta-model strategy enables the system to learn from past data and update its decision model, as opposed to rigid rule-based systems. This characteristic allows the classifier to improve its generalization ability, making its predictions more consistent and reducing the possibility of false positives. Instead of directly weighing or averaging ${AI}_{1}\;$ and ${AI}_{2}\;$ predictions, this method obtains helpful patterns and relationships between their predictions. The meta-classifier will consider previous cases derived from these AI models to function adaptively in selecting the optimum weighting for each provided AI model. The Random Forest algorithm was selected as our primary training procedure for the meta-classifier due to its established efficiency and reliability in predictive analytics [35]. Some of its main strengths are as follows:

- It shows non-linear, messy correlations that may exist between the *AI*_1_ and *AI*_2_ output and thus possibly boost predictive accuracy.
- Unlike logistic regression, which assumes linearity, Random Forest has the capability of handling detailed decision boundaries.
- It builds many decision trees and averages the results, thereby cleaning up overfitting.

**Figure 4:**
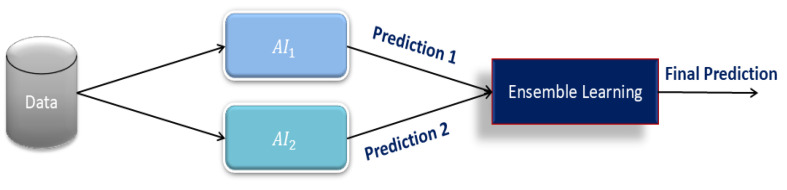
Model of ensemble learning.

This makes the model generalizable to other datasets. As ${AI}_{1}\;$ and ${AI}_{2}\;$ are both capable of generating different risk scores or classes, the Random Forest algorithm automatically determines the input features that contribute significantly to the final prediction. Figure 4 shows the model of ensemble learning. This reduces the need for hand-built features and improves efficiency therein.

Table 4 gives a brief description of the weight given to each risk grade used by ${AI}_{1}\;$ and ${AI}_{2}$. The On-Learning Random Forest meta-classifier will create a data-driven and adaptive older adult fall risk prediction model that is superior to existing fusion methods. The system applies to the principle of ensemble learning to deliver more accurate, interpretable, and scalable older adult fall risk predictions.

### 3.4. Proposed Novel Co-Operative AI-Based Fall Risk Prediction Model Architecture Overview

**Figure 5:**
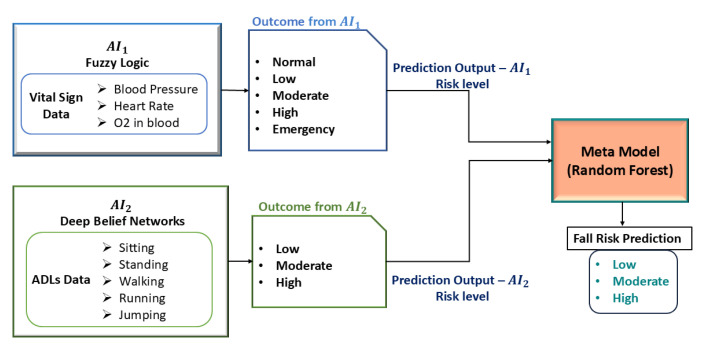
Architecture of the proposed AI-based Cooperative Meta-Model for Fall Risk Prediction.

This paper aims to predict the future risk of falls in the elderly based on the fusion of two significant markers of health: vital signs and ADLs in a cooperative AI-based model for fall risk prediction. The model seeks to maximize predictive accuracy by incorporating a series of artificial intelligence techniques, thereby facilitating timely and accurate estimation of fall risk in close alignment with the MFS, to achieve a very high concordance rate of about 90%. The design consists of two independent AI models, one evaluating vital signs and the other evaluating ADLs, which are then fused into a meta-model via ensemble learning to enhance the overall prediction outcome. The first AI model (${AI}_{1}$) evaluates vital sign patterns using Fuzzy Logic, whereby 111 pre-established fuzzy rules are used to classify fall risk into five categories: Normal (No risk), Low, Moderate, High, and Emergency. In the event of no abnormality detected, the model predicts Normal, which indicates no risk of fall, whereas abnormalities detected are graded according to severity. The second AI model (${AI}_{2}$) uses Deep Belief Networks (DBNs) to analyze ADL patterns and predict fall risk based on learned history and categorizes the risk of falls as Low, Moderate, or High. The ADLs provide important information about mobility, balance, and patterns of movement that allow ${AI}_{2}$ to identify individuals with impaired movement and an elevated risk for falls. Following the predictions by these two models, their output is fed into the meta-model, which is a decision-making model, refining the final prediction. Figure 5 depicts the architecture of the proposed AI-based Cooperative Meta-Model for Fall Risk Prediction.

The meta-model-based fall risk prediction system is a composite model that trains a meta-model classifier, which uses the output of ${AI}_{1}\;$ and ${AI}_{2}\;$ as input features and is utilized to generate a consistent prediction. The Cooperative Meta-Model fall risk prediction algorithm based on the final fall risk prediction is formulated by integrating the output of both ${AI}_{1}\;$ and ${AI}_{2}\;$ models. The meta-model determines ultimate risk levels based on the integrated inputs of ${AI}_{1}$ and ${AI}_{2}$, thus reducing intrinsic errors in individual models and enhancing the trustworthiness of predictions. For instance, if ${AI}_{1}\;\;$ predicts a “*Moderate fall risk*” and ${AI}_{2}\;$ predicts a “*High fall risk*”, the meta-model uses these inputs and calculates the final fall risk outcome based on learned models and training data. This combination-based approach is superior to traditional rule-based models in that it continues to learn from previous predictions and adapts based on new data, thereby increasing its flexibility and credibility. One of the significant benefits of this technique is that it is very accurate, making it possible to be integrated into real-time health monitoring systems. The integration of Fuzzy Logic (${AI}_{1}$) and Deep Belief Networks (${AI}_{2}$) within the meta-model enables intelligent and holistic assessment of the risk of falls based on both physiological markers (vital signs) and behavioral markers (ADLs). The proposed model can be implemented within wearable healthcare technology and home automation systems to allow real-time tracking and immediate fall risk alerts to caregivers and health professionals. The interactive architecture of the system, whereby each AI model enhances the decision-making ability of the meta-model, forms a predictive mechanism that is highly adaptive and specific, learning from new data. The enabling of the meta-model with the ability to compare and harmonize predictions made by AI makes the meta-model a good predictive tool for predicting fall risk among the elderly, thus facilitating early interventions that can significantly reduce the incidence of injuries and the associated healthcare costs resulting from falls. This study illustrates that AI-powered cooperative models are the key to developing predictive healthcare solutions and delivering a scalable and intelligent method for elderly fall risk prediction. This model aims to predict future falls and classify different levels of fall risk in elderly individuals aged 60 and above. Fall risk levels are categorized as Low, Moderate, and High based on the fall risk prediction analysis using the AI-based Cooperative Meta-Model, which informs the prediction outcome. Initially, data from older adults is collected from the public repository for training and testing purposes.

### 3.5. AI-Based Co-Operative Meta-Model Fall Risk Prediction Algorithm

The fall risk prediction algorithm for the AI-based Cooperate Meta-Model is built via a combination of the results created by the ${AI}_{1}\;$ and ${AI}_{2}\;$ models, described in the pseudocode of the algorithm (Algorithm 1). Weighting is performed on every separate model, upon which the final composite risk value is computed. The training labels are set with reference to the combined risk variables alongside the MFS. This is where the meta-model is trained, and the final fall risk prediction is based on learning from the stored data (past data) and the current inputs (present data).
**Algorithm 1.** AI-based Cooperate Meta-Model Fall Risk Prediction Algorithm: Pseudocode of the Proposed Model (Figure 4)Input Mapping:Let:
$\;X_{AI1}$ = Output of ${AI}_{1}\;$ Model—*Normal*, *Low*, *Moderate*, *High*, *Emergency*$\;X_{AI2}$ = Output of ${AI}_{2}\;$ Model—*Low*, *Moderate*, *High*Assign weights $W_{AI1}$ and $W_{AI2}\;$ to the outputs:
                   For $X_{AI1}$: $W_{AI1}=\left\{\begin{array}{c} 0.2\;if\;Normal\\ 0.4\;if\;Low\\ 0.6\;if\;Moderate\\ 0.8\;if\;High\\ 1.0\;if\;Emergency \end{array}\right.$                   For $X_{AI2}$: $W_{AI2}=\left\{\begin{array}{c} 0.4\;if\;Low\\ 0.6\;if\;Moderate\\ 0.8\;if\;High \end{array}\right.$2.Combined Risk Score:The combined risk score $S_{combined}$ is calculated as: $\;\;\;\;\;\;\;\;\;\;\;\;\;\;\;\;\;\;\;\;\;\;\;\;\;\;\;\;\;\;\;\;\;\;\;\;\;\;S_{combined}$ = $\frac{W_{AI1}+W_{AI2}}{2}$
3.Define Risk Levels:Risk level *R* is defined based on $S_{combined}$:                   $R=\left\{\begin{array}{c} Low\;if\;0\leq \;S_{combined}\leq 0.40\\ Moderate\;if\;0.41\leq \;S_{combined}\leq 0.80\\ High\;0.81\leq \;S_{combined}\leq 1.00 \end{array}\right.$
4.Train Random Forest Meta-ModelLet the dataset consist of
Training features $X_{train}=\{W_{AI1},\;W_{AI2}\}$
Training labels $Y_{train}$ = MFS-based risk levels {Low, Moderate, High}
5.Random Forest Model:Initialize *T*, the number of decision trees. n_estimators = 100 max_depth = 10 min_samples_split = 4 For each tree *t* in *T:*
Select a random subset $X_{t}$ from $X_{train}$Build a decision tree using $X_{t}$ and $Y_{train}$Split data at each node using features $\left\{W_{AI1},\;W_{AI2}\right\}$Aggregate predictions from all *T* trees by majority voting.
6.Predict Fall Risk:For testing data $X_{test}=\{W_{AI1},\;W_{AI2}\}$
Pass each test instance through all *T* decision trees.
Compute the predicted risk level $R_{pred}\;$ based on a majority vote from the *T* trees.
7.Output ResultsFor each participant *i*, output: Participant i: {$S_{combined}$,$R_{pred}$}

## 4. Results

This section evaluates the performance of the proposed AI-based Cooperative Meta-Model for Fall Risk Prediction according to its data acquisition process and experimental results. Its accuracy is calculated based on the correctness of its predictions in comparison to the Morse Fall Scale. The simulations were executed using Visual Studio Code IDE on Windows 10 Enterprise, based on an 11th Gen Intel Core i7 processor (3.00 GHz) with 32 GB RAM.

### 4.1. Evaluation Metrics

The proposed framework is evaluated using data from PhysioNet [36], which includes

- Age;
- History of falls;
- Vital sign data;
- ADL data.

The Confusion Matrix is used as an evaluation approach for fall risk prediction. These measurements are accuracy, sensitivity, and specificity, with TP, TN, FP, and FN representing True Positive, True Negative, False Positive, and False Negative, respectively [20]. The result analysis integrates data from both the ${AI}_{1}\;$ and ${AI}_{2}\;$ models to ensure a comprehensive assessment of fall risk prediction. However, due to the unavailability of a dataset containing both vital signs and ADLs from the same participants, an indirect approach was adopted. In this method, two different participants with the same age, a similar history of falls, and comparable health conditions were selected, one from the ${AI}_{1}\;$ model database and the other from the ${AI}_{2}$ model database. To help guarantee consistency, both participants also shared the same MFS score. For the meta-model, these two participants were merged as a single entity to simulate the presence of both vital signs and ADL data. Following such a systematic process, a training dataset of 30 participants was established, while another 15 participants were held out for testing. The performance of the proposed model was evaluated by comparing its prediction with the MFS to determine its accuracy and reliability. The MFS scoring system categorizes the risk of falls into three groups: Low risk (below 25), Moderate risk (between 25 and 45), and High risk (above 45). To attain compatibility with the scoring system utilized in the proposed AI-based model, the MFS scores were converted into their equivalent numerical values: Low risk (0.3), Moderate risk (0.6), and High risk (0.9). This step allowed for a homogeneous and standardized evaluation, whereby a direct comparison of the AI-based Cooperative Meta-Model predictions with the traditional MFS-based fall risk prediction could be made. Through this method, the proposed model demonstrates its ability to provide valid and accurate fall risk prediction in the elderly.

### 4.2. Evaluation of the Developed AI-Based Meta-Model with Morse Falls Scale (MFS)

The process of result evaluation integrates information from the ${AI}_{1}\;$ and ${AI}_{2}\;$ models to provide a comprehensive overview of fall risk assessment [24,27]. The key components for the same, i.e., age of participants, history of falls, vital signs, and Activities of Daily Living (ADLs), were considered with care so that uniformity and reliability were maintained while evaluating. For creating a representative dataset, 10 participants from the ${AI}_{1}\;$ model database and 10 participants from the ${AI}_{2}\;$ model database with matching ages, history of falls, and medical history were selected. This procedure was adopted to ensure that the selected individuals possess comparable health status to perform a fair and systematic evaluation. Since each dataset contained either vital sign data (${AI}_{1}$) or ADL data (${AI}_{2}$), an indirect pairing approach was employed, where participant P1 of ${AI}_{1}\;$ was matched with participant P1 of ${AI}_{2}$, forming a single participant 1 to be assessed. This was applied consistently to all the selected participants, essentially combining the two different data dimensions into a single evaluation dataset. As a result, the total number of participants to be involved in the testing phase was narrowed down to 10. The full comparative analysis of the Cooperative Meta-Model and the MFS is given in Table 5, comparing the results of classification obtained from both frameworks.

**Figure 6:**
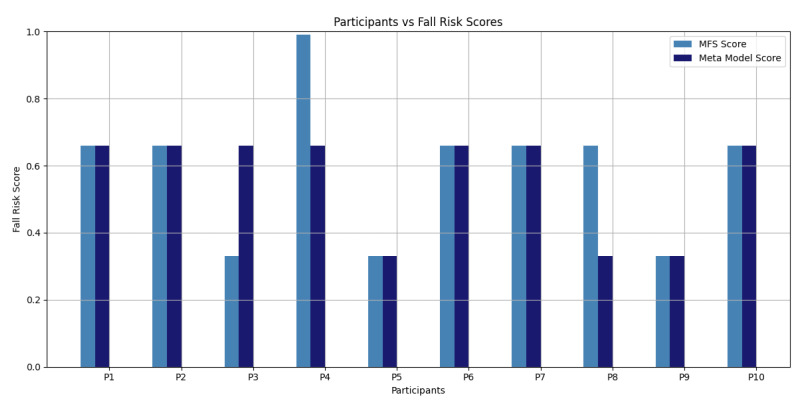
Fall risk prediction evaluation results.

The proposed AI-based model allocated participants P5, P8, and P9 with “*low fall risk*”, whereas other participants were placed in the “*moderate fall risk*” category. By comparison, the MFS outcomes positioned participants P3, P5, and P9 in “*low risk of fall*”, P4 in “*high risk of fall*”, with the rest in “*moderate risk of fall*”. A comparison of the Cooperative Meta-Model and MFS predictions showed a high correlation, indicating the model’s good predictive strength. There were, however, discrepancies in the case of P3, P4, and P8, where model predictions were different from the MFS classifications. Such discrepancies can be due to factors such as the variability in participants’ fall history over the last year or the small size of the training dataset used for AI model development. Figure 6 provides a graphical comparison of the simulation results of the Cooperative Meta-Model and the MFS-based risk assessment. In comparison to the MFS, the Cooperative Meta-Model was found to have a remarkable accuracy of 90.00%, a sensitivity of 85.75%, and a specificity of 100% with TP (6), TN (3), FP (0), and FN (1). The results confirm the effectiveness and reliability of the proposed model as a predictor of falls in older adults, highlighting its viability for practical application in proactive fall prevention.

## 5. Discussion

This paper provided the motivational foundation for designing an AI-based cooperative fall risk prediction system and its feasibility in dynamically assessing and responding to potential fall risks among older adults. Based on the problem space, the most significant building blocks necessary for developing a feasible, adaptive, and proactive model for fall prediction are critically examined with a focus on two aspects: smart risk assessment and continuous health monitoring. The study finds the inclusion of crucial vital signs and behavior information to develop a robust predictive model that effectively recognizes individuals at risk, thus permitting early intervention for preventing falls and enhancing care among the elderly.

The first part refers to the proposed fall risk prediction model, on the principles of Fuzzy Logic, which has been thoughtfully created to determine the at-risk older adults for falling. The main reason for the model development was to create a valid and useful model for the prediction of falls, thereby assisting in reducing the economic load and personal difficulties of fall-related injuries among older adults. Via ongoing monitoring of vital signs, the model is linked to three important physiological parameters, such as blood pressure, heart rate, and blood oxygenation, to detect early deviations that may indicate a higher risk of falls. Unlike existing approaches relying on a mix of multiple health parameters, our model is distinguished by its single reliance on vital signs, making it a small yet efficient fall risk prediction model. Regarding the MFS and using three sources of data, the model was 95.24% accurate with a 100% specificity and 93.75% sensitivity. These values show the model’s efficiency and consistency in classifying fall risk levels with virtually no false positives or false negatives. Different research works on fall risk assessment highlight that most elderly falls are preventable by responding at the earliest possible time. Even with the excellent accuracy of the proposed ${AI}_{1}$ model, the model in question has not yet been validated on big real-time series data.

Adding real-time continuous observation of older persons is required to strengthen the model and its forecast. The biggest factor remaining to be validated is the level of the emergency risk indicator that can be validated empirically separately. Acquiring real-time tests and data may introduce new versions that could impact the prescribed fuzzy rules, thereby requiring additional adjustments. The second section is regarding the suggested fall risk prediction model based on a DBN, specifically for the elderly market and aimed at detecting risks of falls by tracking behavioral patterns in real-time, especially for some ADLs. The model predicts fall risk based solely on behavior data. The model’s accuracy is 91.67%, specificity is 100%, and sensitivity is 90%, and it can therefore correctly classify at-risk patients and non-at-risk patients. A major limitation of this study is that the model only considers four ADLs out of the five that need to be considered. Even though it had been trained on an enormous database for 75 h of watching, the lack of jumping behavior failed to give the utmost prediction potential to achieve high accuracy. Thus, the model achieved less than 95% accuracy, indicating that inclusion of the missing ADL might have an impact on the prediction result: a further increase in accuracy or a lowering of it below 90%. Hence, the effect of such inclusion is unforeseeable. To resolve this, future research will entail expanding the dataset by including all five ADLs and further validation with real-time tracking. This will determine if the expanded dataset gives higher predictive reliability.

One key limitation of this study is the use of an indirect pairing method to combine ADL data with vital signs, as fully synchronized multi-modal datasets were not available. Although we carefully selected matching records from PhysioNet’s eICU and Long-term Movement Database, this approach may introduce distribution bias across subjects. Furthermore, only 45 subjects (30 for training and 15 for testing) met our inclusion criteria, which may limit the statistical generalizability of the findings. Due to time, budget, and the absence of an ethics application, we were constrained to publicly available data. The current model evaluation does not include k-fold or hold-out cross-validation due to the limited sample size. We recognize these factors may affect model robustness and plan to address them through larger datasets and cross-validation in future work. Despite these constraints, the study offers a foundational approach that can be further refined.

## 6. Conclusions and Future Work

In short, the Cooperative Meta-Model for AI-based Fall Risk Prediction is a significant breakthrough in the prevention of geriatric care. In contrast to traditional fall-detecting systems that react only after a fall has happened, the model offers an anticipation model by real-time checking of vital signs and Activities of Daily Living (ADLs) to investigate the risk of falls in real-time. With the integration of Fuzzy Logic for physiological assessment (${AI}_{1}$) and Deep Belief Networks for behavioral assessment (${AI}_{2}$) in a meta-learning paradigm, the model possesses superior prediction efficiency over time, demonstrated through 90.00% accuracy, 100% specificity, and 85.75% sensitivity. The results indicate the ability of the model to accurately ascertain those truly in danger without the incidence of false positives and false negatives. Going forward, future work aims to complement this system with the development of a non-invasive, continuous monitoring solution that seamlessly becomes a part of daily life for older adults. This will be the third AI model employing transfer learning to combine outcomes from the enhanced ${AI}_{1}$ and ${AI}_{2}$ models into a single unified, personalized fall risk score. As part of future work, the main aim is to address this limitation detailed under Section 5, Discussion, by utilizing larger datasets and implementing appropriate cross-validation techniques. Nonetheless, this study provides a foundational framework that can be further enhanced by employing transfer learning. While the proposed fall risk prediction model demonstrates promising accuracy, its current design does not support real-time execution on wearable devices. The wearable system is intended solely for data acquisition, with all processing and analysis performed in the cloud. This cloud-based architecture avoids placing computational strain on the device but introduces potential latency that may affect time-sensitive interventions. Real-time prediction, especially on edge devices with limited processing power, remains a challenge. Future research will focus on optimizing the model for lightweight deployment and exploring edge AI frameworks to enable real-time decision-making, balancing accuracy with low-latency performance. The cooperative and adaptive nature of this multi-model system facilitates continuous learning and synchronization of the physiological and behavioral indicators, enabling early detection of pending health risks. In the end, this new paradigm offers data-driven, scalable technology that prioritizes prevention, maximizes safety, and improves the quality of life of the elderly.

## Figures and Tables

**Table 1:** Weight assignment for ${AI}_{1}$ and ${AI}_{2}$ models.

${AI}_{1}$	Score	${AI}_{2}$	Score
Normal	0.2	Low	0.33
Low	0.4		
Moderate	0.6	Moderate	0.66
High	0.8	High	0.99
Emergency	1.0		

**Table 2:** Score fusion metrics.

Comparator	Score
Low	0.0–0.40
Moderate	0.41–0.80
High	0.81–1.00

**Table 3:** Decision tree approach predictions.

${AI}_{1}$	${AI}_{2}$	Final Prediction (Decision Tree)
Low	Low	Low
Moderate	High	High
Emergency	Any	Emergency
Moderate	High	Moderate–High (Weighted Decision)
Low	Moderate	Low-Moderate (Adjusted Based on Condition)

**Table 4:** Weights assigned to each risk level for ${AI}_{1}$ and ${AI}_{2}\;$ models.

${AI}_{1}$	Score	${AI}_{2}$	Score
Normal	0.2	Low	0.4
Low	0.4		
Moderate	0.6	Moderate	0.6
High	0.8	High	0.8
Emergency	1.0		

**Table 5:** Comparative result analysis based on our proposed model and MFS prediction.

Participant	Fall History	Input		Output: Prediction Results	
		${AI}_{1}$	${AI}_{2}$	Co-Operative Meta-Model Prediction	MFS Prediction
P1	2 (Y)	Moderate	Low	Moderate	Moderate
P2	2 (Y)	Moderate	Low	Moderate	Moderate
P3	0 (N)	Moderate	Low	Moderate	Low
P4	5 (Y)	Moderate	Moderate	Moderate	High
P5	0 (N)	Normal	Low	Low	Low
P6	6 (Y)	High	Moderate	Moderate	Moderate
P7	2 (Y)	High	Low	Moderate	Moderate
P8	1 (Y)	Low	Low	Low	Moderate
P9	0 (N)	Low	Low	Low	Low
P10	2 (Y)	Moderate	Moderate	Moderate	Moderate

**Figure A1:**
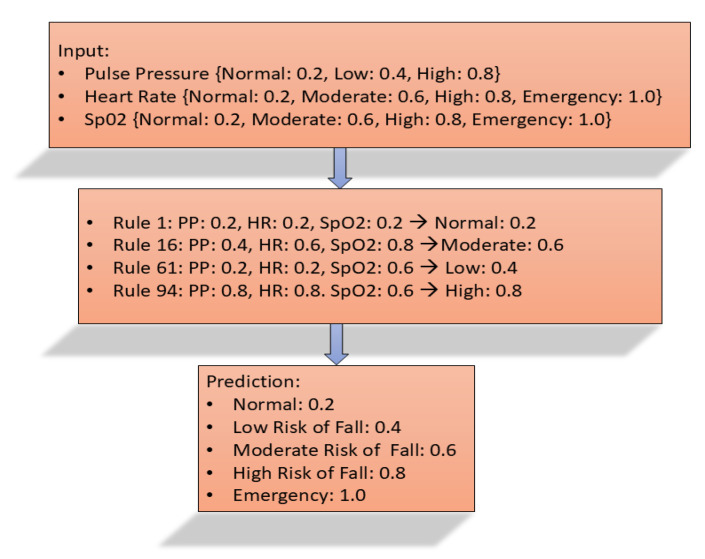
Fuzzy rule evaluation metrics [20].

**Figure A2:**
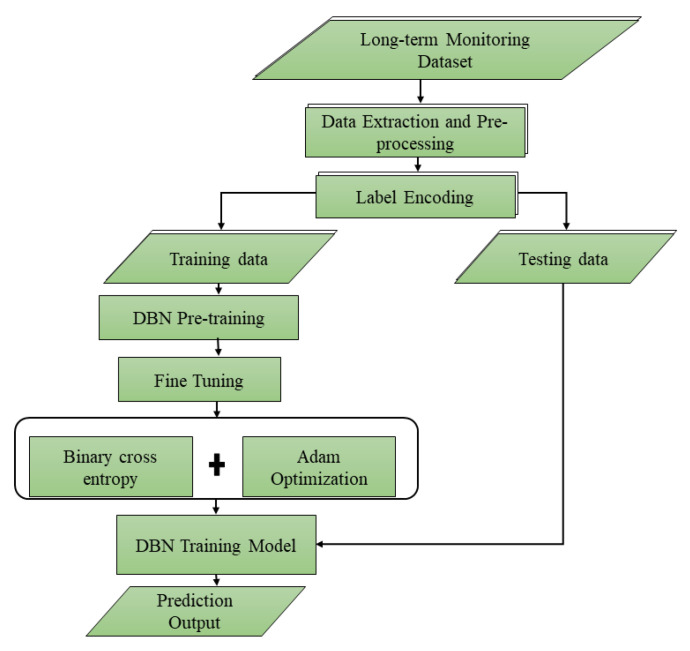
Workflow structure of the developed DBN model [26].

## Data Availability

Datasets obtained from the Physionet public repository.

## Appendix A

### Appendix A.1

The ${AI}_{1}$ model in this research predicts falls in older adults using a fuzzy rule-based fall prediction algorithm (FPA) [20]. It monitors vital signs, such as blood pressure, heart rate, and blood oxygen levels, and classifies fall risk into five levels: Normal, Low, Moderate, High, and Emergency.

- Blood Pressure: The first sign of health issues is often abnormal blood pressure, either high or low.
- Heart Rate*:* A change in blood pressure can affect heart rate, causing it to rise or fall from the normal 60–80 bpm range.
- Blood Oxygen (O_2_): Fluctuations in heart rate may lower blood oxygen levels below the normal 95–98% in older adults.

Although blood pressure (BP) and heart rate (HR) are not directly linked, changes in BP can influence HR in older adults. Table A1 presents the overall vital metrics chart used in this research. Based on background analysis and expert advice, the vital sign patterns are thoroughly examined and devised 111 fuzzy rules for this research to determine the level of future fall risk in an elderly person, and the risk levels were classified as follows:

- Normal: No risk of fall.
- Low: May or unlikely to experience a fall.
- Moderate: Risk of fall in the future if this condition persists.
- High: High chances of falling and to seek medical help if necessary.
- Emergency: Very high chances of fall (fall will occur in a few seconds or minutes)—alert messages will be sent to the caregiver, family, and emergency services.

**Table A1:** Vital metrics for ${AI}_{1}$ model [20].

BP (mmHg)		Normal	Low Risk	Moderate Risk	High Risk		Emergency
Normal BP	SV/DV	100/60–120/80	-	-	-		-
Low BP	SV/DV	90/60–70/40					
High BP	SV/DV	121/80–190/100					
PP	SV-DV	40–60	<40 and >60				
HR (bpm)		Normal		Moderate Risk	High Risk (73–85% max HR)		Emergency (93% max HR)
Age	60–70	60–100		101–109	110–139		>140, <60
	71–80	60–100		101–102	103–129		>130, <60
	81–90	60–80		81–94	95–119		>120, <60
	91–100	60–80		81–87	88–109		>110, <60
${SpO}_{2}$ (%)		Normal		Moderate Risk		High Risk	Emergency
Age	60–100	97–100		95–96		90–94	<90

The fall prediction algorithm (Algorithm A1) is based on a set of vital sign data, as defined in the algorithm (FPA) pseudocode. The chosen datasets are based on age, fall history, and observed vital sign values.

**Algorithm A1.** Fall Prediction Algorithm (FPA): Pseudocode of the Proposed Model [20]
**Input:** *Blood Pressure*, *Heart Rate and*$\;O_{2}\;$*in Blood*—Datasets from public Repository. **Output***: Fall Prediction Outcome*—*Risk level classification* 1. Apply data sets for data extraction to the input datasets. 2. Feed the retrieved features to the fuzzy prediction model to train it and formulate rules for categorizing risk levels. (a) Fuzzification: Transform crisp data into fuzzy data. (b) Membership function: Formulate the membership function based on the parameter values (*Normal*, *Low*, *Moderate*, *High*, *and Emergency*) Input function*:* ‘trapmf’. (c) Fuzzy Rule Generation: Formulate fuzzy rules from the fuzzy data. Fuzzy rule: Rule 1: For Normal BP, if PP is Normal and HR is Normal and $O_{2}$ is Normal *then* Risk Level is Normal (No risk of fall detected) .Rule 83: For High BP, (126–130/80–90) if PP is High and HR is High (110–129 bpm) and $O_{2}$ is Moderate (95–96%) then Risk Level is High (Will have a risk of falling in few days if this condition persists) (d) Defuzzification: Transform the fuzzy rules into crisp rules. 3. Employ cross-validation on the model using the testing dataset with MFS. Evaluate the model’s performance based on accuracy, specificity, and sensitivity metrics. 4. Perform evaluation results to validate the results.

The membership function (MF) ranges from 0 to 1, with each fall risk level assigned a specific weight: Normal—0.2, Low—0.4, Moderate—0.6, High—0.8, and Emergency—1.0. For example, if the BP is 90/60, the systolic (S_v) and diastolic (D_v) values give a pulse pressure (PP) of 30. Based on this, a sample prediction rule is as follows:

- IF BP is 90/60, PP < 40, HR = 102, and *O*_2_ = 95, THEN fall risk = 0.6.
- IF BP is 120/80, PP = 40, HR = 90, and *O*_2_ = 97, THEN fall risk = 0.2.

The fuzzy membership function converts these numerical “IF” conditions into linguistic variables (e.g., LOW, MODERATE, HIGH). For instance,

- IF BP is HIGH; PP is LOW; HR is MODERATE; and *O*_2_ is MODERATE; THEN fall risk is MODERATE.

All numerical decision rules are first processed, converted into fuzzy rules using the MF, and then evaluated using the FPA. Finally, defuzzification is applied to generate fall risk categories. Figure A1 illustrates this fuzzy rule evaluation process.

### Appendix A.2

Based on prior research on monitoring abnormal behavior in the elderly, the importance of Activities of Daily Living (ADLs) in fall prediction is well established [26]. This study focuses on five key ADLs: sitting, standing, walking, running, and jumping.

The model was trained using ADL datasets from older adults aged 65 to 87, obtained from PhysioNet’s Long-term Monitoring Database [36]. A total of 71 participants wore 3D accelerometers for continuous activity monitoring over 3 days (approximately 75 h). However, only 66 recordings were available in the repository. In this research, data from 50 participants with 65–75 h of recordings were used for training, and data from 12 participants were used for testing. Four subjects were excluded due to recordings being under 30 h.

Participants were categorized into two groups [26]:

- Fallers (Fx): More than two falls in a year;
- Non-fallers/Control Group (Cx): Zero or fewer than two falls annually. Details of the participant data used in this study are summarized in Table A2.

Each participant’s dataset in this proposed research has over 10,000 records from the 3D accelerometer sensor. Data conversion is carried out following the procedure given in [37], which specifies how to identify ADLs from raw recordings. These research-specific ADLs, namely sitting, standing, walking, running, and jumping, were then employed to train and test the built DBN model. Fallers’ and non-fallers’ data were gathered for every 10 min interval (10 min × 60 min each hour × 75 h over three days) and then converted into comparable ADLs. This pre-processed data serves as the input for the developed AI-2 model. An explanation of the ADL conversion algorithm is given below.

ADL Data Extraction: This research utilizes ADL data to predict falls, which is derived from the raw data collected from a 3D accelerometer placed on the lower back of each participant to monitor gait. The raw recordings are then processed using an ADL conversion algorithm (Algorithm A2) that extracts meaningful activity classifications based on predefined threshold values.

**Table A2:** Description of participants’ recordings—PhysioNet [26].

Data Analysis	Participant Count
Total elderly participant data as mentioned in PhysioNet	71
Total records found	66
Usable participants dataset for research	62
Data with missed recordings that are not usable	4
Total training data used	50 (Fx-21, Cx-29)
Total testing data used	12 (Fx-7, Cx-5)

**Algorithm A2.** ADL Conversion Algorithm—Pseudocode [26]
**Input:** 3D Accelerometer Raw Data **Output:** ADL (Sitting, Standing, Walking, Running, Jumping) and Unknown **Initialization:** CLOCK_FREQUENCY=100 ticks/second WINDOW_SIZE=100 Data Frame à df Time Parsing: For each entry in the ‘Time (elapsed)’ column Parse_time (h:m:s) = h x 3600 + m x 60 + s Remove NaN rows. Feature Extraction: For each sliding window of size n from df: mean_v = $\frac{1}{n}\sum_{i=1}^{n}v_{acceleration}[i]$ mean_ml = $\frac{1}{n}\sum_{i=1}^{n}{ml}_{acceleration}[i]$ mean_ap = $\frac{1}{n}\sum_{i=1}^{n}{ap}_{acceleration}[i]$ std_v = $\sqrt{\frac{1}{n-1}\sum_{i=1}^{n}(v_{acceleration}\left[i\right]{-mean\_v)}^{2}}$ std_ml = $\sqrt{\frac{1}{n-1}\sum_{i=1}^{n}({ml}_{acceleration}\left[i\right]{-mean\_ml)}^{2}}$ std_ap = $\sqrt{\frac{1}{n-1}\sum_{i=1}^{n}({ap}_{acceleration}\left[i\right]{-mean\_ap)}^{2}}$ **Activity Classification:** - Sitting - Standing - Walking - Running - Jumping

The proposed Deep Belief Network (DBN) model consists of three Restricted Boltzmann Machine (RBM) layers: RBM_1, RBM_2, and RBM_3—with 256, 200, and 150 hidden units, respectively. These values were optimized through iterative trials to maximize accuracy. Each RBM is trained using contrastive divergence in a bottom-up, layer-by-layer (greedy) approach, as outlined in [18]. After training, the output of each RBM serves as the input to the next.

Input weights are based on fall risk-related activities:

- Sitting: 0.1
- Standing: 0.2
- Walking: 0.3
- Running: 0.6
- Jumping: 0.8
- History of Falls (Yes = 1, No = 0)

Risk categories are weighted as

- Low risk: 0.3
- Moderate risk: 0.6
- High risk: 0.9

Each RBM layer captures higher-order features through unsupervised learning. After pre-training, the learned weights are used to initialize a deep neural network, forming a stacked DBN (Algorithm A3). During supervised fine-tuning, earlier layers may be frozen to retain learned features, and the model is trained on labeled fall risk data using the Adam Optimizer. Only unfrozen layers are updated, and a softmax classifier produces final risk outputs scaled to Low (0.3), Moderate (0.6), or High (0.9).

**Algorithm A3.** DBN-based Fall Prediction Algorithm (DBN-FPA): Pseudocode of the Proposed Model [26]
**Input:** *ADLs*, *Falls History*—Datasets from public Repository. **Output*:****Risk level classification—Fall Prediction Outcome* 1. Data extraction from the input parameters 2. *Feature extraction* → Feed extracted data to the DBN-based prediction model. (a) Data Normalization - (X) → input data with features (fall history, ADLs). - Min-Max normalization to each feature in (X): $\;X^{\prime }=\frac{X-X_{min}}{X_{max}-X_{min}}$ where $X_{min}$, and $X_{max}$ → minimum and maximum values of X (b) Encode Labels - Y → target labels using label encoding (c) Split normalized data → X’ encoded labels → Y data into training and test sets (d) Pre-train with RBMs - Train RBM_1: - components=256, learning_rate=0.01, _iter=10 - Train RBM_2: - components=200, learning_rate=0.01, _iter=10 - Train RBM_3: - components=150, learning_rate=0.01, _iter=10 (e) Fine-tuning → Neural Network + Adam Optimizer (f) Initialize the weights of the neural network with the weights from the pre-trained RBMs: - Model_layers [0] → set_weights → RBM_1 - Model_layers [1] → set_weights → RBM_2 - Model_layers [2] → set_weights → RBM_3 3. Train using DBN- Risk Calculation 4. Cross-validation using testing dataset → Determine the accuracy, specificity, and sensitivity of the model. 5. Result Evaluation → validate the findings.

Backpropagation enables deep networks to learn complex patterns and plays a significant role in building deep learning models, namely, convolutional and recurrent neural networks.

- **RBM_1 (First Hidden Layer–Low-Level Features)**

*Input:* Raw data (Sitting, Standing, Walking, Running and Jumping).

*Output:* Extracts low-level features from the input.

- **RBM_2 (Second Hidden Layer–Mid-Level Features)**

*Input:* Features learned by RBM_1.

*Output:* Recognizes higher-order relationships between features.

- RBM_3 (Third Hidden Layer–High-Level Features)

*Input:* Features learned by RBM_2.

*Output:* Finds complex patterns in the dataset.

The workflow structure of the developed DBN model is shown in Figure A2.
